# Impact of extranodal involvement at CAR T-cell therapy on outcomes in patients with relapsed or refractory large B-cell lymphoma—Results from a multicenter cohort study

**DOI:** 10.1038/s41408-025-01318-5

**Published:** 2025-06-21

**Authors:** Frederique St-Pierre, Subodh Bhatta, Peter G. Doukas, Madeline Jenkin, Kaitlin Annunzio, Alexandra E. Rojek, Alyssa Gibson, Yun Kyoung Tiger, Brittany McCall, Khaled Alhamad, Alec Hansen, Juan P. Alderuccio, Olutobi Adewale, Keem Patel, Asaad Trabolsi, Izidore S. Lossos, Lindsey Fitzgerald, Thomas A. Ollila, Matthew J. Matasar, Justin Kline, Reem Karmali, Narendranath Epperla

**Affiliations:** 1 Great River Health/Southeast Iowa Regional Medical Center, Department of Hematology/Oncology, West Burlington, IA USA; 2 The James Cancer Hospital and Solove Research Institute, The Ohio State University, Department of Medicine, Division of Hematology, Columbus, OH USA; 3 Robert H. Lurie Comprehensive Cancer Center, Northwestern University, Division of Hematology/Oncology, Chicago, IL USA; 4 Feinberg School of Medicine, Northwestern University, Chicago, IL USA; 5 University of Chicago, Department of Medicine, Section of Hematology/Oncology, Chicago, IL USA; 6 Rutgers Cancer Institute of New Jersey, Division of Blood Disorders, New Brunswick, NJ USA; 7 Rhode Island Hospital, Division of Hematology-Oncology, Providence, RI USA; 8 Division of Hematology and Hematologic Malignancies, Huntsman Cancer Institute, University of Utah, Salt Lake City, UT USA; 9 University of Utah, Department of Medicine, Salt Lake City, UT USA; 10 University of Miami Miller School of Medicine, Division of Hematology, Department of Medicine, Miami, FL USA

**Keywords:** B-cell lymphoma, B-cell lymphoma, Cancer immunotherapy, Disease-free survival

## Abstract

Extranodal (EN) diffuse large B-cell lymphoma (DLBCL) has been historically associated with inferior survival outcomes compared to nodal DLBCL. However, outcomes of patients with EN DLBCL following chimeric antigen receptor T-cell (CAR-T) therapy are not well established. In this multi-center retrospective cohort study, we evaluated the outcomes of patients with EN DLBCL who underwent CAR-T in the relapsed/refractory (R/R) setting. The primary objective was overall survival (OS), while secondary objectives included progression-free survival (PFS), response rates, and toxicity rates. A total of 218 patients were included in the analysis. The most common sites of EN involvement were skin/soft tissue (25%), bone (22%), and lung (17%). Overall response rate (ORR) and complete response rate (CRR) at first post-treatment evaluation were 62% (*n* = 127) and 40% (*n* = 82), respectively. Median follow-up was 3.5 years. Median PFS and OS were 4.0 months (95% CI = 3.1–7.2) and 25.7 months (95% CI = 16.1–51.6), respectively. Cytokine release syndrome (CRS) of any grade occurred in 73% (*n* = 159) of patients, and 6% (*n* = 12) had grade ≥ 3 CRS. Immune effector cell-associated neurotoxicity syndrome (ICANS) of any grade occurred in 37% (*n* = 81) of patients, and 19% (*n* = 41) developed grade ≥ 3 ICANS. In the multivariable analysis, factors that were independently prognostic of inferior OS were 3 or more lines of therapy prior to CAR-T, bulky disease at the time of CAR-T, hepatobiliary, and pancreas involvement, while refractory disease to the most recent therapy prior to CAR-T was associated with inferior PFS. Future studies should further evaluate outcomes of CAR-T in patients with specific EN sites of involvement that appear to be associated with inferior survival such as the liver and pancreas.

## Introduction

Extranodal (EN) lymphoma is defined by involvement of sites outside of the lymph nodes, spleen, thymus and oropharyngeal lymphoid tissue [1]. Up to 50% of patients with diffuse large B-cell lymphoma (DLBCL) have EN involvement at diagnosis [2, 3]. The most common sites of EN disease in DLBCL are the gastrointestinal tract, bone, bone marrow, and skin/soft tissue [3, 4]. EN DLBCL is distinct from nodal DLBCL in its molecular pathogenesis, clinical presentation, and prognosis [5, 6]. In addition, EN lymphoma is a highly heterogeneous disease, and biologic features and prognosis vary significantly based on the specific site of EN involvement [3, 6–8]. Low prevalence of each EN site has resulted in scarce data to inform best practices and optimal management in these patients [6].

Chimeric antigen receptor (CAR) T-cell therapy has revolutionized the treatment landscape of relapsed or refractory (R/R) DLBCL with approval in second line for primary refractory and early relapse as well as in the third line [9–14]. Prior studies evaluating the outcomes of EN DLBCL receiving CAR-T have been limited in study size and have shown conflicting findings on the impact of EN disease at time of CAR-T on outcomes [15, 16]. We therefore sought to evaluate outcomes and toxicities in patients with EN DLBCL undergoing CAR-T in the R/R setting using a large, multi-institutional database.

## Patients and methods

### Study Design

This is a multicenter retrospective cohort study of patients with EN R/R large B-cell lymphoma (LBCL) receiving CAR T-cell therapy from 8 US academic centers between January 1, 2016, and March 1, 2024. To be eligible, R/R LBCL patients must have received anti-CD19 CAR T-cell therapy. Patients with active EN involvement (either primary or secondary EN disease) at the time of apheresis were included. Patients with primary central nervous system (CNS) lymphoma were excluded, while those with secondary CNS lymphoma were excluded from the main (primary) analysis but were included in the secondary analysis. Patients who had EN disease at diagnosis but not at the time of CAR-T (*n* = 25) were excluded from the primary analysis and were only included in the exploratory analysis.

### Study objectives and definitions

The primary objective was to evaluate the overall survival (OS) of patients with EN R/R LBCL undergoing CAR-T. Secondary objectives included progression-free survival (PFS), response rates (overall response rate [ORR] and complete response rate [CRR]) to CAR-T, and CAR-T toxicities including cytokine release syndrome (CRS), immune effector cell-associated neurotoxicity syndrome (ICANS), prolonged (>28 days post CAR-T infusion) need for transfusion of packed red blood cells (PRBCs) or platelets, and prolonged (>28 days post CAR-T infusion) need for granulocyte stimulating agents.

In this study, we retrospectively identified patients with LBCL and included patients with any subtype of DLBCL and high-grade B-cell lymphoma (HGBCL), as well as transformed indolent lymphoma histologies. Patients with disease that arises from an EN site are deemed to have primary EN lymphoma, while patients with disease arising from a nodal site and spreading to an EN site have secondary EN disease. OS was defined as the time from CAR-T infusion to death from any cause, and PFS was calculated from CAR-T infusion to radiologic or symptomatic disease progression, death from any cause, with censoring for those alive at the time of last follow up. ORR was defined as the proportion of patients achieving either a partial or complete response to CAR T-cell therapy, as determined by institutional radiologic response criteria on the first post-treatment positron emission tomography/computed tomography (PET/CT). CRR was defined as the proportion of patients showing no evidence of disease on the first post-treatment PET/CT, based on institutional radiologic response criteria.

### Ethics approval and consent to participate

The study was approved by the institutional review board at Northwestern University and at all participating sites and was conducted in compliance with the Declaration of Helsinki. As this was a retrospective study, informed consent was waived.

### Statistical analysis

Descriptive statistics were used to describe the demographic and disease characteristics. Median and range were used to summarize continuous variables, whereas categorical variables were described using frequency and percentages. PFS and OS were estimated using the Kaplan-Meier method. The Cox proportional hazards models were used to evaluate hazard ratios associated with the risk of progression or death. The univariable analysis for both OS and PFS was conducted using the same set of variables, which included all EN sites and other clinically important variables. Variables found to be significant in the univariable analysis were included in the multivariable analysis. Exploratory analyses were performed for other variables outside of those predetermined for univariable and multivariable models. Analyses were performed using R version 4.3.2, and all the estimates were reported with 95% confidence interval (CI).

## Results

### Patient characteristics

A total of 218 patients were included in the analysis (see Consort diagram, Figure S1). Baseline characteristics of the cohort are outlined in Table 1. The median age at diagnosis was 62 years (range: 20–90), with 64% (*n* = 140) males. At the time of CAR-T, 74% (*n* = 161) of the patients had DLBCL, 19% (*n* = 41) had high-grade B-cell lymphoma (HGBCL), and 7% (*n* = 16) had transformed indolent lymphoma. The median follow up time was 3.50 years (range: 0.08–6.99).

**Table 1:** Baseline patient characteristics.

Variable	*N* = 218 (%)
**Age (years) at CAR-T, median, range**	62 (20–90)
**Gender**	
Male	140 (64)
Female	78 (36)
**Race**	
White	175 (82)
Black/African American	19 (9)
Other	20 (9)
**Ethnicity**	
Non-Hispanic	200 (93)
Hispanic/Latino	15 (7)
**Primary refractory disease after first line therapy (*n*** = **177)**	
No	88 (58)
Yes	65 (42)
**Number of lines of therapy prior to CAR-T**	
1-2	106 (49)
≥3	111 (51)
**Auto-HCT prior to CAR-T**	43 (20)
**XRT prior to CAR-T**	32 (21)
**Primary vs secondary EN involvement at the time of CAR-T (*n*** = **151)**	
Primary	56 (37)
Secondary	95 (63)
**Number of EN sites involved at the time of CAR-T**	
1	134 (62)
≥2	83 (38)
**Histology at the time of CAR T**	
DLBCL	161 (74)
Transformed indolent lymphoma^a^	16 (7)
HGBCL	41 (19)
**Stage at last relapse prior to CAR-T**	
I–II	12 (6)
III–IV	203 (94)
**Age-adjusted IPI at last relapse prior to CAR-T**	
0–1	75 (36)
2–3	134 (64)
**ECOG PS at time of CAR-T**	
0–1	201 (97)
≥2	6 (3)
**Bulky disease (≥7 cm) at last response prior to CAR-T (*n*** = 149)	
No	97 (76)
Yes	31 (24)
**Refractory to most recent therapy prior to CAR-T**	
No	58 (28)
Yes	146 (72)
**Bridging therapy prior to CAR-T**	
No	100 (46)
Yes	118 (54)
**CAR-T product**	
Axi-cel	99 (45)
Tisa-cel	61 (28)
Liso-cel	58 (27)
**EN site involved**	
Skin/Soft tissue	55 (25)
Bone	48 (22)
Lung/Pleura	38 (17)
GI tract	31 (14)
Bone marrow	31 (14)
Hepatobiliary	26 (12)
Kidney	11 (5)
Pancreas	8 (4)
Orbit/Sinus/Nasopharynx	9 (4)
Adrenal gland	6 (3)
Breast	2 (1)
Heart/Pericardium	2 (1)
**Median follow-up in years (range) from CAR-T**	3.50 (0.08-6.99)

*Axi-cel* Axicabtagene ciloleucel, *CAR-T* Chimeric antigen receptor T-cell therapy, *DLBCL* Diffuse large B-cell lymphoma, *ECOG* Eastern Cooperative Oncology Group, *EN* Extranodal, *GI* Gastrointestinal, *HGBCL* High-grade B-cell lymphoma, *IPI* International prognostic index, *Liso-cel* Lisocabtagene maraleucel, *PS* Performance status, *Tisa-cel* Tisagenlecleucel, *W/O* without, *XRT* radiation therapy.

^a^Includes all transformed indolent histologies except Richter’s transformation.

### Sites of EN involvement

**Fig. 1:**
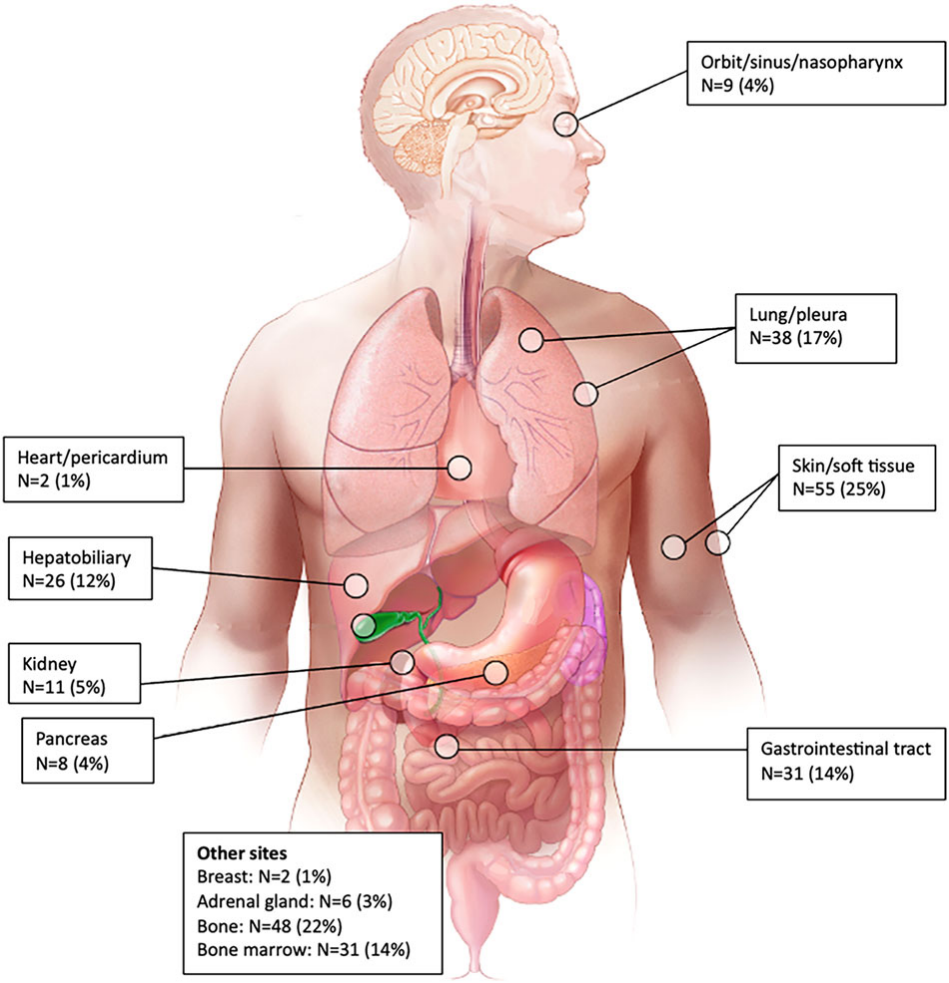
Distribution of extranodal sites at the time of CAR-T. Permission granted by Wiley to reproduce figure from: 10.1002/ajh.25493.

At the time of CAR-T, 62% (*n* = 134) of patients had 1 EN site involved, and 38% (*n* = 83) of patients had presence of two or more EN sites. The most common site involved was skin/soft tissue in 25% (*n* = 55) of patients, followed by bone (22%, *n* = 48) and lung (17%, *n* = 38) involvement. Other common sites involved included the gastrointestinal (GI) tract, bone marrow and liver. Distribution of EN sites involved at the time of CAR-T is illustrated in Fig. 1. Outcomes by site of EN involvement are outlined in Table S1.

### Response rates and Toxicities

ORR and CRR at first post-treatment evaluation were 62% (*n* = 127) and 40% (*n* = 82), respectively. Response rates are detailed in Table S2.

CRS of any grade occurred in 73% (*n* = 159) of patients, and 6% (*n* = 12) had grade ≥ 3 CRS. The median duration of CRS was 4 days (range: 1–57). ICANS of any grade occurred in 37% (*n* = 81) of patients, and 19% (*n* = 41) developed grade ≥ 3 ICANS. The median duration of ICANS was 5 days (range: 1–188).

Prolonged need for PRBC or platelet transfusion occurred in 19% (*n* = 39) and 20% (*n* = 42) of the patients, respectively. Prolonged need for granulocyte colony-stimulating factor (G-CSF) occurred in 36% (*n* = 75) of patients. A summary of CAR-T toxicities is outlined in Table 2.

**Table 2:** Summary of CAR-T toxicities in patients with R/R EN LBCL.

Variable	*N* = 218 (%)
**CRS of any grade**	159 (73)
Grades 1–2	147 (67)
Grades ≥3	12 (6)
**Median duration of CRS in days (range)**	4 (0-57)
**ICANS of any grade**	81 (37)
Grades 1–2	40 (18)
Grades ≥3	41 (19)
**Median duration of ICANS in days (range)**	5 (0-188)
**Prolonged PRBC transfusion needs (>28 days)**	39 (19)
**Prolonged platelet transfusion needs (>28 days)**	42 (20)
**Prolonged filgrastim needs (>28 days)**	75 (36)
**Secondary malignancy after CAR-T**	5 (3)

*CAR-T* Chimeric antigen receptor T-cell therapy, *CRS* cytokine release syndrome, *EN* Extranodal, *ICANS* Immune effector cell-associated neurotoxicity syndrome, *LBCL* Large B-cell lymphoma, *PRBC* packed red blood cells, *R/R* Relapsed/refractory.

### Progression-free survival

**Fig. 2:**
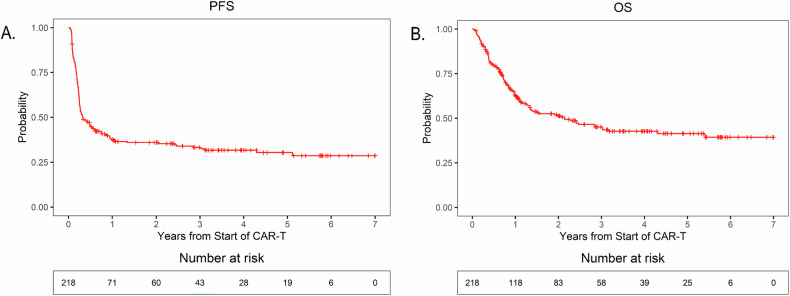
Progression-free survival and overall survival in patients with extranodal relapsed/refractory large B-cell lymphoma undergoing CAR-T. **A** PFS probability by year from the start of CAR-T. **B** OS probability by year from the start of CAR-T.

The median PFS was 4.0 months (95% CI = 3.1–7.2). The 1-, 3-, and 5-year PFS estimates were 38% (95% CI = 32–45), 33% (95% CI = 27–40), and 30% (95% CI = 24–38), respectively (Fig. 2A).

In the univariable analysis, factors associated with significantly inferior PFS included age-adjusted IPI ≥ 2, bulky disease at the time of CAR-T, defined as a tumor measuring ≥ 7 centimeters (cm), refractory disease to the most recent therapy prior to CAR-T, use of bridging therapy prior to CAR-T, and EN disease involving the liver/biliary tract (Table S3). In the multivariable analysis, refractory disease to the most recent therapy prior to CAR-T remained associated with significantly inferior PFS, with a hazard ratio (HR) of 2.55 (95% CI = 1.36–4.79, *p* < 0.01, Table S3).

### Overall survival

The median OS was 25.7 months (95% CI = 16.1–51.6). The 1-, 3-, and 5-year OS estimates were 63% (95% CI = 56–70), 45% (95% CI = 38–53), and 41% (95% CI = 35–50), respectively (Fig. 2B).

In the univariable analysis, factors associated with significantly inferior OS included ≥ 3 lines of therapy prior to CAR-T, age-adjusted IPI ≥ 2, bulky disease at the time of CAR-T (≥7 cm), refractory disease to the most recent therapy prior to CAR-T, use of bridging therapy prior to CAR-T, EN involvement of the liver/biliary tract, and pancreas (Table 3). After controlling for these factors in the multivariable analysis, patients receiving ≥ 3 lines of therapy prior to CAR-T (HR = 2.20, 95% CI = 1.28–3.78, *p* < 0.01), bulky disease at the time of CAR-T (HR = 2.56, 95% CI = 1.40–4.66, *p* = 0.01), liver/biliary tract involvement (HR = 2.05, 95% CI = 1.01–4.15, *p* = 0.05) and pancreas involvement (HR = 6.2, 95% CI = 2.20–17.46, *p* < 0.01) remained independently prognostic of inferior OS (Table 3).

**Table 3:** Univariable and multivariable analysis for overall survival.

Variable	OS			
	Univariable		Multivariable	
	HR (95% CI)	*p*-value	HR (95% CI)	*p*-value
**Gender**				
Male	1			
Female	1.0 (0.68, 1.49)	0.99		
**Number of lines of therapy prior to CAR-T**				
0–2	1		1	
≥3	1.56 (1.06, 2.27)	**0.02**	2.11 (1.23, 3.61)	**<0.01**
**Histology at time of CAR-T**				
DLBCL	1			
Transformed lymphoma	0.94 (0.47, 1.86)	0.86		
HGBCL	0.64 (0.37, 1.1)	0.10		
**Ann Arbor stage at last relapse prior to CAR-T**				
I–II	1			
III–IV	0.91 (0.40, 2.06)	0.81		
**Number of EN sites involved at the time of CAR-T**				
1	1			
≥2	1.41 (0.97, 2.05)	0.07		
**Bulky disease (≥7** **cm) at the time of CAR-T**				
No	1		1	
Yes	2.28 (1.36, 3.82)	**<0.01**	2.65 (1.46, 4.79)	**<0.01**
**Age-adjusted IPI at CAR-T**				
0–1	1		1	
2–3	1.69 (1.11, 2.57)	**0.01**	1.17 (0.65, 2.11)	0.61
**Refractory to most recent therapy prior to CAR-T**				
No	1		1	
Yes	1.86 (1.15, 3.02)	**0.01**	1.77 (0.89, 3.50)	0.10
**Bridging therapy prior to CAR-T**				
No	1		1	
Yes	1.83 (1.27, 2.65)	**<0.01**	1.68 (0.96, 2.97)	0.07
**CAR-T product**				
Axi-cel	1			
Tisa-cel	1.65 (0.93, 2.92)	0.43		
Liso-cel	0.87 (0.56, 1.44)	0.65		
**Hepatobiliary involvement**				
No	1		1	
Yes	2.57 (1.58, 4.48)	**<0.01**	1.98 (0.99, 3.97)	**0.05**
**Pancreas involvement**				
No	1			
Yes	2.35 (1.03, 5.37)	**0.04**	6.50 (2.35, 17.93)	**<0.01**

*Axi-cel* Axicabtagene ciloleucel, *CAR-T* Chimeric antigen receptor T-cell therapy, *DLBCL* Diffuse large B-cell lymphoma, *EN* Extranodal, *HGBCL* High-grade B-cell lymphoma, *HR* Hazard ratio, *IPI* International prognostic index, *LDH* lactate dehydrogenase, *Liso-cel* Lisocabtagene maraleucel, *OS* Overall survival, *Tisa-cel* Tisagenlecleucel.

Bold value signifies statistical significance.

### Outcomes for patients with EN disease including secondary CNS involvement who underwent CAR-T

As secondary CNS involvement accounts for the highest risk EN disease, we performed secondary analysis of patients with EN involvement including those with secondary CNS lymphoma (*n* = 236) who underwent CAR-T.

CRS of any grade occurred in 74% (*n* = 175) of patients and 9% (*n* = 15) had grade ≥ 3 CRS. ICANS of any grade occurred in 38% (*n* = 90) of patients, and 19% (*n* = 45) developed grade ≥ 3 ICANS. The rates of CRS and ICANS were similar to that seen in patients with EN disease without secondary CNS involvement. A summary of CAR-T toxicities for patients with EN disease who underwent CAR-T based on secondary CNS involvement are outlined in Table S4. The response rates (ORR and CRR) were comparable for patients with EN disease who underwent CAR-T regardless of the secondary CNS involvement (see Table S5).

The median PFS was 4.0 months (95% CI = 3.1–7.2). The 1-, 3-, and 5-year PFS estimates were 38% (95% CI = 32–44), 32% (95% CI = 26–39), and 29% (95% CI = 23-36), respectively (Figure S2A). The median OS was 23.8 months (95% CI = 16.0–37.9). The 1-, 3-, and 5-year OS estimates were 62% (95% CI = 56–68), 44% (95% CI = 38–52), and 41% (95% CI = 34–49), respectively (Figure S2B). These survival estimates were comparable to patients with EN disease who underwent CAR-T without secondary CNS involvement.

### Exploratory analysis

We considered whether patients who had EN disease at initial diagnosis but no longer had EN disease at the time of apheresis would have different outcomes from patients who had active EN disease at apheresis (Table S6). An exploratory analysis comparing these patient groups showed no differences in toxicity (Table S7) or efficacy outcomes (Table S8 and Figures S3).

## Discussion

In this multi-institutional retrospective cohort study, we evaluated the outcomes of patients with R/R LBCL receiving CAR T-cell therapy and made several important observations. First, the ORR and CRR were 62% and 40%, respectively. Second, the rates of grade ≥3 CRS and ICANS were 6% and 19%, respectively. Third, refractory disease to the most recent therapy prior to CAR-T was associated with significantly inferior PFS in the multivariable analysis. Lastly, we found that 3 or more lines of therapy prior to CAR-T, bulky disease at the time of CAR-T, hepatobiliary, and pancreas involvement were independently associated with significantly inferior OS.

Previous real-world studies evaluating outcomes associated with CAR-T in R/R LBCL found ORR and CRR ranging between 60-80% and 30-65%, respectively [17–20], which is comparable to the response rates noted in our study. Similarly, the median PFS following CAR-T in our study was in line with the published data (3-9 months) [17–20], however, the median OS post-CAR-T was slightly longer in our study (25.7 months) relative to the prior studies (12–22 months) [17–20]. There was a higher proportion of patients in our study that had received 1–2 lines of therapy prior to CAR-T versus 3 or more prior lines of therapy, which may account for this difference in OS. Of note, most of these real-world studies did not provide information regarding the proportion of patients with EN LBCL versus nodal LBCL in their cohort.

In prior studies, the most common toxicities post CAR-T included CRS, ICANS, and prolonged cytopenias [17–20]. Rate of CRS typically ranged between 55-85%, with 5-10% developing grade ≥ 3 CRS. Rate of ICANS ranged between 30–55%, with 5–25% developing grade ≥ 3 ICANS. Prolonged cytopenia (most commonly neutropenia and thrombocytopenia) occurred in 10–25% of patients [17–20]. In line with these studies, we found a comparable rate of all-grade and grade ≥ 3 CRS (73% and 6%, respectively), all-grade and grade ≥ 3 ICANS (37% and 19%, respectively) and prolonged need for blood count support (18% for PRBC transfusions, 20% for platelet transfusions, 36% for G-CSF) in our study.

A few retrospective studies have compared the outcomes of EN DLBCL and nodal DLBCL following CAR-T. One retrospective study of 37 patients with R/R DLBCL treated with CAR-T (*n* = 19 patients with EN DLBCL, *n* = 18 patients with nodal DLBCL) showed that one-year PFS and OS were inferior in patients with EN DLBCL compared to nodal DLBCL (PFS = 83.3% vs. 42.1%, *p* = 0.008; OS = 94.4% vs. 63.2%, *p* = 0.020) [15]. This study had a small sample size and limited median follow up of 13 months. A separate study evaluated 126 patients receiving CAR-T for R/R DLBCL in patients with EN DLBCL (*n* = 72) versus nodal DLBCL (*n* = 52) and showed similar median PFS (10.8 vs. 14.1 months, *p* = 0.126) and OS (15.4 vs. 18.4 months, *p* = 0.10) between the two groups [16]. A third retrospective study of 47 patients who underwent CAR-T for R/R LBCL (*n* = 25 patients with EN DLBCL, *n* = 22 patients with nodal DLBCL) showed no statistically significant difference in OS between patients with versus without EN disease at the time of CAR-T [21]. A fourth recently published study of 516 patients with R/R LBCL (*n* = 177 patients with only nodal disease, *n* = 66 patients with only EN disease, *n* = 273 patients with both nodal and EN disease) showed no outcome difference between patients with nodal versus EN disease, although patients with both nodal and EN involvement had shorter PFS and OS [22]. Our results support the findings of the latter studies suggesting similar outcomes to CAR-T in patients with EN R/R LBCL compared to nodal LBCL, as the PFS, OS, and toxicity rates in our cohort were comparable to those traditionally described in the literature for R/R LBCL.

We identified several risk factors in patients with EN R/R LBCL undergoing CAR-T that were independently prognostic of inferior OS, including three or more lines of therapy prior to CAR-T and bulky disease at the time of CAR-T. The site of EN involvement is also important, as evidenced by our findings that involvement of the liver/biliary tract and pancreas at the time of CAR-T portended poor OS. Findings from a recently published study detailed above (*n* = 516) also indicated poorer outcomes (shorter PFS) in patients with liver and pancreas involvement [22]. We postulate that different sites of involvement may be associated with distinct tumor microenvironments, with some features resulting in additional mechanisms of resistance to CAR-T.

Of note, given the poor prognosis associated with CAR-T for patients with secondary CNS involvement [4, 23–25], we performed additional analysis evaluating the outcomes of patients with EN disease with secondary CNS involvement who underwent CAR-T. The toxicity and efficacy outcomes in this cohort were found to be comparable in those without secondary CNS involvement. One potential reason for this might be related to the small sample size of the patients with secondary CNS lymphoma who underwent CAR-T (*n* = 18) in the current study.

Our study is limited by the inherent limitations of the retrospective study design. The molecular and clinical characteristics of primary versus secondary disease are distinct from each other [6], however, due to sample size limitations we did not have sufficient statistical power to evaluate the differences in outcomes between primary and secondary EN LBCL. Given the relatively small sample size of each individual EN site, we were not able to further analyze trends regarding patient/disease characteristics within these subgroups. We could not analyze the outcomes based on nodal and extranodal versus extranodal only given the way data was collected. Lastly, the study was not designed to compare the outcomes of those with and without EN disease at CAR-T and the results of the exploratory analysis should be interpreted as such.

## Conclusions

In this large, multi-institutional study evaluating the outcomes of patients with EN R/R LBCL undergoing CAR-T, the response rates, survival outcomes and the rate of high-grade CRS and ICANS were comparable to the real-world outcomes of CAR-T in R/R LBCL. Future studies should further evaluate outcomes of CAR-T in patients with specific EN sites of involvement that appear to be associated with inferior survival such as the liver and pancreas.

## Supplementary information

Supplementary Appendix

## Data Availability

Data is available upon request to the corresponding author as permitted by the IRB.

## Third party material

Permission granted by Wiley to reproduce Fig. 1 from: 10.1002/ajh.25493.
