# Supplementary material for: Impact of extranodal involvement at CAR T-cell therapy on outcomes in patients with relapsed or refractory large B-cell lymphoma—Results from a multicenter cohort study
Source: Blood Cancer J. 2025 Jun 21;15(1):110. doi: 10.1038/s41408-025-01318-5 (PMC12182562; doi:10.1038/s41408-025-01318-5)
Supplement: Supplementary file 1 — Supplementary Appendix [file 41408_2025_1318_MOESM1_ESM.docx]

**SUPPLEMENTAL APPENDIX**

**Table of Contents** 1

**Outcomes by site of EN involvement**: Table S1 2-3

**Response rates to CAR-T in patients with EN R/R LBCL**: Table S2 4

**Univariable and multivariable analysis for PFS**: Table S3 5-6

**Summary of CAR-T toxicities based on secondary CNS involvement:** Table S4 7

**Response rates in patients with and without secondary CNS disease:** Table S5 8

**Baseline characteristics based on EN disease at CAR-T:** Table S6 9-10

**Summary of CAR-T toxicities by status of EN involvement at CAR-T:** Table S7 11

**Response rates in patients without EN disease at CAR-T:** Table S8 12

**Consort diagram:** Figure S1 13

**PFS and OS of pts with EN dz including SCNSL:** Figure S2 14

**PFS and OS by status of EN involvement at the time of CAR-T:** Figure S3 15

**Table S1. Outcomes by site of EN involvement**

| **Variable** | **PFS (HR, 95%CI)** | **p-value** | **OS (HR, 95%CI)** | **p-value** | **CRS of any grade (%)** | **p-value** | **ICANS of any grade (%)** | **p-value** |
| --- | --- | --- | --- | --- | --- | --- | --- | --- |
| **Number of EN sites involved** |  |  |  |  |  |  |  |  |
| 0-1 | 1 |  | 1 |  | 72.39 |  | 36.57 |  |
| >=2 | 1.23 (0.88, 1.71) | 0.23 | 1.41 (0.97, 2.05) | 0.07 | 74.70 | 0.83 | 38.55 | 0.88 |
| **GI tract** |  |  |  |  |  |  |  |  |
| No | 1 |  | 1 |  | 73.26 |  | 37.43 |  |
| Yes | 0.93 (0.58, 1.47) | 0.75 | 0.99 (0.58, 1.72) | 0.99 | 70.97 | 0.96 | 35.48 | 0.99 |
| **Lung/Pleura** |  |  |  |  |  |  |  |  |
| No | 1 |  | 1 |  | 73.33 |  | 40.56 |  |
| Yes | 0.85 (0.55, 1.31) | 0.47 | 0.81 (0.50, 1.34) | 0.42 | 71.05 | 0.93 | 21.05 | **0.04** |
| **Kidney** |  |  |  |  |  |  |  |  |
| No | 1 |  | 1 |  | 73.43 |  | 37.20 |  |
| Yes | 1.06 (0.52, 2.15) | 0.88 | 0.99 (0.46, 2.13) | 0.98 | 63.64 | 0.49 | 36.36 | 1.00 |
| **Breast** |  |  |  |  |  |  |  |  |
| No | 1 |  | 1 |  | 72.69 |  | 36.57 |  |
| Yes | 0.51 (0.07, 3.62) | 0.50 | NA (NA, NA)* | NA | 100 | 1.00 | 100.00 | 0.14 |
| **Pancreas** |  |  |  |  |  |  |  |  |
| No | 1 |  | 1 |  | 74.29 |  | 38.57 |  |
| Yes | 1.95 (0.86, 4.42) | 0.11 | 2.35 (1.03, 5.37) | **0.04** | 37.50 | **0.04** | 100.00 | **0.03** |
| **Heart/Pericardium** |  |  |  |  |  |  |  |  |
| No | 1 |  | 1 |  | 73.61 |  | 37.5 |  |
| Yes | 1.42 (0.35, 5.73) | 0.63 | 2.12 (0.52, 8.61) | 0.30 | 0 | 0.07 | 0 | 0.53 |
| **Skin/Soft tissue** |  |  |  |  |  |  |  |  |
| No | 1 |  | 1 |  | 72.39 |  | 36.20 |  |
| Yes | 0.81 (0.55, 1.19) | 0.29 | 0.73 (0.46, 1.16) | 0.18 | 74.55 | 0.89 | 40.00 | 0.73 |
| **Bone** |  |  |  |  |  |  |  |  |
| No | 1 |  | 1 |  | 73.53 |  | 37.06 |  |
| Yes | 1.05 (0.71, 1.54) | 0.81 | 1.16 (0.76, 1.78) | 0.50 | 70.83 | 0.85 | 37.50 | 1.00 |
| **Adrenal gland** |  |  |  |  |  |  |  |  |
| No | 1 |  | 1 |  | 72.64 |  | 36.79 |  |
| Yes | 1.43 (0.59, 3.51) | 0.43 | 2.41 (0.98, 5.94) | 0.06 | 83.33 | 1.00 | 50.00 | 0.67 |
| **Hepatobiliary** |  |  |  |  |  |  |  |  |
| No | 1 |  | 1 |  | 72.40 |  | 36.98 |  |
| Yes | 1.81 (1.14, 2.88) | **0.01** | 2.57 (1.58, 4.48) | **< 0.01** | 76.92 | 0.80 | 38.46 | 1.00 |
| **Nasopharynx/ Orbit/Sinus** |  |  |  |  |  |  |  |  |
| No | 1 |  | 1 |  | 73.68 |  | 37.80 |  |
| Yes | 1.42 (0.66, 3.03) | 0.37 | 0.75 (0.28, 2.03) | 0.60 | 55.56 | 0.26 | 22.22 | 0.49 |
| **Bone marrow** |  |  |  |  |  |  |  |  |
| No | 1 |  | 1 |  | 71.66 |  | 36.36 |  |
| Yes | 1.09 (0.69, 1.73) | 0.71 | 1.42 (0.87, 2.30) | 0.16 | 80.65 | 0.41 | 41.94 | 0.69 |

CRS: Cytokine release syndrome; EN: Extranodal; GI: Gastrointestinal; HR: Hazard ratio; ICANS: Immune effector cell-associated neurotoxicity syndrome; OS: Overall survival; PFS: Progression free survival

*Infinitely small HR, due to small sample size.

**Table S2. Response rates* to CAR-T in patients with R/R EN LBCL**

| **Variable** | **N=218* (%)** |
| --- | --- |
| ORR | 127 (62) |
| CRR | 82 (40) |
| PR | 45 (22) |
| SD | 15 (7) |
| PD | 62 (31) |

CAR-T: Chimeric antigen receptor T-cell therapy; CRR: Complete response rate; EN: Extranodal; LBCL: Large B-cell lymphoma; ORR: Overall response rate; PD: Progressive disease; PR: Partial response; R/R: Relapsed/refractory; SD: Stable disease.

*Response rates are based on first post-treatment positron emission tomography/computed tomography (PET/CT); hence, the n is 204.

**Table S3. Univariable and multivariable analysis for progression-free survival**

|  | **Univariable** | | **Multivariable** | |
| --- | --- | --- | --- | --- |
| **Variable** | **HR (95% CI)** | **p-value** | **HR (95% CI)** | **p-value** |
| **Gender** |  |  |  |  |
| Male | 1 |  |  |  |
| Female | 0.73 (0.51, 1.03) | 0.07 |  |  |
| **Number of lines of therapy prior to CAR-T** |  |  |  |  |
| 1-2 | 1 |  |  |  |
| ≥3 | 1.36 (0.98, 1.89) | 0.07 |  |  |
| **Histology at time of CAR-T** |  |  |  |  |
| DLBCL | 1 |  |  |  |
| Transformed lymphoma | 1.06 (0.60, 1.88) | 0.84 |  |  |
| HGBCL | 0.70 (0.44, 1.11) | 0.13 |  |  |
| **Ann Arbor stage at last relapse prior to CAR-T** |  |  |  |  |
| I-II | 1 |  |  |  |
| III-IV | 0.98 (0.46, 2.09) | 0.95 |  |  |
| **Age-adjusted IPI at CAR-T** |  |  |  |  |
| 0-1 | 1 |  | 1 |  |
| 2-3 | 1.87 (1.28, 2.71) | < 0.01 | 1.47 (0.89, 2.45) | 0.14 |
| **Number of EN sites at CAR-T** |  |  |  |  |
| 1 | 1 |  |  |  |
| ≥2 | 1.23 (0.88, 1.71) | 0.23 |  |  |
| **Bulky disease (≥7cm) at CAR-T** |  |  |  |  |
| No | 1 |  | 1 |  |
| Yes | 1.6 (1, 2.56) | 0.05 | 1.50 (0.90, 2.50) | 0.13 |
| **Refractory to most recent therapy prior to CAR-T** |  |  |  |  |
| No | 1 |  | 1 |  |
| Yes | 1.99 (1.3, 3.04) | < 0.01 | 2.55 (1.36, 4.79) | < 0.01 |
| **Bridging therapy prior to CAR-T** |  |  |  |  |
| No | 1 |  | 1 |  |
| Yes | 1.44 (1.03, 2) | 0.03 | 1.24 (0.77, 1.99) | 0.38 |
| **CAR-T product** |  |  |  |  |
| Axi-cel | 1 |  |  |  |
| Tisa-cel | 0.81 (0.55, 1.19) | 0.29 |  |  |
| Liso-cel | 0.74 (0.49, 1.12) | 0.16 |  |  |
| **Hepatobiliary involvement*** |  |  |  |  |
| No | 1 |  | 1 |  |
| Yes | 1.81 (1.14, 2.88) | 0.01 | 1.53 (0.84, 2.78) | 0.17 |
| **Pancreas involvement*** |  |  |  |  |
| No | 1 |  |  |  |
| Yes | 1.95 (0.86, 4.42) | 0.11 |  |  |

Axicabtagene ciloleucel; CAR-T: Chimeric antigen receptor T-cell therapy; DLBCL: Diffuse large B-cell lymphoma; EN: Extranodal; HGBCL: High-grade B-cell lymphoma; HR: Hazard ratio; IPI: International prognostic index; Liso-cel: Lisocabtagene maraleucel; PFS: Progression-free survival; Tisa-cel: Tisagenlecleucel.

*Only the EN sites with statistical significance in PFS or OS are included. For details pertaining to individual EN site, please refer to Table S1

**Table S4. Summary of CAR-T toxicities for patients with EN disease who underwent CAR-T based on the secondary CNS involvement**

|  | **EN disease including secondary CNS involvement**  **n=236 (%)** | **EN disease not including secondary CNS involvement**  **n=218 (%)** |
| --- | --- | --- |
| **CRS of any grade** | 175 (74) | 159 (73) |
| Grades 1-2 | 160 (68) | 147 (67) |
| Grades 3-4 | 15 (6) | 12 (6) |
| **ICANS of any grade** | 90 (38) | 81 (37) |
| Grades 1-2 | 44 (19) | 40 (18) |
| Grades 3-4 | 45 (19) | 41 (19) |

CAR-T: Chimeric antigen receptor T-cell therapy; CRS: cytokine release syndrome; EN: Extranodal; ICANS: Immune effector cell-associated neurotoxicity syndrome

**Table S5. Response rates* in patients patients with EN disease who underwent CAR-T based on the secondary CNS involvement**

|  | **EN disease including secondary CNS involvement**  **n=236* (%)** | **EN disease not including secondary CNS involvement**  **n=218* (%)** |
| --- | --- | --- |
| **ORR** | 137 (63) | 127 (62) |
| **CR** | 88 (41) | 82 (40) |
| **PR** | 49 (23) | 45 (22) |
| **SD** | 15 (7) | 15 (7) |
| **PD** | 65 (30) | 62 (30) |

ORR: Overall response rate; PD: Progressive disease; PR: Partial response; R/R: Relapsed/refractory; SD: Stable disease.

*Response rates are based on first post-treatment positron emission tomography/computed tomography (PET/CT); hence, the n for EN disease with SCNL is 217 and the n for EN disease excluding SCNSL is 204.

**Table S6. Baseline patient characteristics based on presence or absence of EN disease at the time of CAR-T**

| **Variable** | **All**  **N=243 (%)** | **EN at CAR-T**  **N=218 (%)** | **W/O EN at CAR-T N=25 (%)** | **P value** |
| --- | --- | --- | --- | --- |
| **Age (years) at CAR-T, median, range** | 63 (20-90) | 62 (20-90) | 65 (42-82) | 0.16 |
| **Gender** |  |  |  |  |
| Male | 151 (62) | 140 (64) | 11 (44) | 0.08 |
| Female | 92 (38) | 78 (36) | 14 (56) |  |
| **Race** |  |  |  |  |
| White | 199 (83) | 175 (82) | 24 (96) | 0.25 |
| Black/African American | 19 (8) | 19 (9) | 0 (0) |  |
| Other | 21 (9) | 20 (9) | 1 (4) |  |
| **Ethnicity** |  |  |  |  |
| Non-Hispanic | 222 (93) | 200 (93) | 22 (88) | 0.41 |
| Hispanic/Latino | 18 (7) | 15 (7) | 3 (12) |  |
| **Primary refractory disease after first line therapy (n=177)** |  |  |  |  |
| No | 101 (57) | 88 (58) | 13 (54) | 0.93 |
| Yes | 76 (43) | 65 (42) | 11 (46) |  |
| **Number of lines of therapy prior to CAR-T** |  |  |  |  |
| 0-2 | 120 (50) | 106 (49) | 14 (56) | 0.64 |
| ≥3 | 122 (50) | 111 (51) | 11 (44) |  |
| **Auto-HCT prior to CAR-T** | 50 (21) | 43 (20) | 7 (28) | 0.49 |
| **Consolidative XRT prior to CAR-T** | 35 (20) | 32 (21) | 3 (12) | 0.42 |
| **Histology at the time of CAR T** |  |  |  |  |
| DLBCL | 181 (74) | 161 (74) | 20 (80) | 0.07 |
| Transformed indolent lymphoma | 20 (8) | 16 (7) | 4 (16) |  |
| HGBCL | 42 (17) | 41 (19) | 1 (4) |  |
| **Stage at last relapse prior to CAR-T** |  |  |  |  |
| I-II | 15 (6) | 12 (6) | 3 (12) | 0.18 |
| III-IV | 224 (94) | 203 (94) | 21 (88) |  |
| **Age-adjusted IPI at last relapse prior to CAR-T** |  |  |  |  |
| 0-1 | 86 (37) | 75 (36) | 11 (46) | 0.46 |
| 2-3 | 147 (63) | 134 (64) | 13 (54) |  |
| **ECOG PS at time of CAR-T** |  |  |  |  |
| 0-1 | 224 (97) | 201 (97) | 23 (100) | 1.00 |
| ≥2 | 6 (3) | 6 (3) | 0 (0) |  |
| **Bulky disease (≥7cm) at last response prior to CAR-T (n=149)** |  |  |  |  |
| No | 116 (78) | 97 (76) | 19 (90) | 0.17 |
| Yes | 33 (22) | 31 (24) | 2 (10) |  |
| **Refractory to most recent therapy prior to CAR-T** |  |  |  |  |
| No | 64 (28) | 58 (28) | 6 (26) | 1.00 |
| Yes | 163 (72) | 146 (72) | 17 (74) |  |
| **Bridging therapy prior to CAR-T** |  |  |  |  |
| No | 115 (47) | 100 (46) | 15 (60) | 0.26 |
| Yes | 128 (53) | 118 (54) | 10 (40) |  |
| **CAR-T product** |  |  |  |  |
| Axi-cel | 107 (44) | 99 (45) | 8 (32) | 0.41 |
| Tisa-cel | 69 (28) | 61 (28) | 8 (32) |  |
| Liso-cel | 67 (28) | 58 (27) | 9 (36) |  |
| **Median follow-up in years (range) from CAR-T** | 3.50 (0.08- 6.99) | 3.50 (0.08-6.99) | 3.32 (0.30-4.83) | 0.12 |

Axi-cel: Axicabtagene ciloleucel; CAR-T: Chimeric antigen receptor T-cell therapy; DLBCL: Diffuse large B-cell lymphoma; ECOG: Eastern Cooperative Oncology Group; EN: Extranodal; HGBCL: High-grade B-cell lymphoma; IPI: International prognostic index; Liso-cel: Lisocabtagene maraleucel; PS: Performance status; Tisa-cel: Tisagenlecleucel; W/O: without; XRT: radiation therapy.

**Table S7. Summary of CAR-T toxicities by status of EN involvement at the time of CAR-T**

| **Variable** | **All**  **N=243 (%)** | **EN at CAR-T**  **N=218 (%)** | **No EN at CAR-T**  **N=25 (%)** | **P-value** |
| --- | --- | --- | --- | --- |
|  | **N (%)** | **N (%)** | **N (%)** |  |
| **CRS of any grade** | 176 (72) | 159 (73) | 17 (68) | 0.77 |
| Grade 1-2 | 163 (67) | 147 (67) | 16 (64) | 0.90 |
| Grade ≥3 | 13 (5) | 12 (6) | 1 (4) | 1.00 |
| **Median duration of CRS in days (range)** | 3 (0-57) | 4 (0-57) | 3 (1-5) | 0.09 |
| **ICANS of any grade** | 89 (37) | 81 (37) | 8 (32) | 0.77 |
| Grade 1-2 | 44 (18) | 40 (18) | 4 (16) | 1.00 |
| Grade ≥3 | 45 (19) | 41 (19) | 4 (16) | 1.00 |
| **Median duration of ICANS in days (range)** | 5 (0-188) | 5 (0-188) | 6 (0-13) | 0.32 |
| **Prolonged PRBC transfusion needs (> 28 days)** | 42 (18) | 39 (19) | 3 (13) | 0.78 |
| **Prolonged platelet transfusion needs (> 28 days)** | 47 (20) | 42 (20) | 5 (22) | 0.79 |
| **Prolonged filgrastim needs**  **(> 28 days)** | 81 (35) | 75 (36) | 6 (26) | 0.48 |
| **Secondary malignancy after CAR-T** | 5 (3) | 5 (3) | 0 (0) | 1.00 |

CAR-T: Chimeric antigen receptor T-cell therapy; CRS: cytokine release syndrome; EN: Extranodal; ICANS: Immune effector cell-associated neurotoxicity syndrome; PRBC: packed red blood cells.

**Table S8. Response rates in patients without EN disease at the time of CAR-T versus with EN disease at CAR-T**

| **Variable** | **EN at CAR-T**  **N=218 (%)** | **W/O EN at CAR-T**  **N=25 (%)** |
| --- | --- | --- |
| ORR | 127 (62) | 18 (72) |
| CR | 82 (40) | 13 (52) |
| PR | 45 (22) | 5 (20) |
| SD | 15 (7) | 2 (8) |
| PD | 62 (31) | 5 (20) |

CAR-T: Chimeric antigen receptor T-cell therapy; CRR: Complete response rate; EN: Extranodal; LBCL: Large B-cell lymphoma; ORR: Overall response rate; PD: Progressive disease; PR: Partial response; R/R: Relapsed/refractory; SD: Stable disease.

**Figure S1. Consort diagram**

^These patients (n=18) were included in the secondary analysis

*These patients (n=25) were compared to those with EN at CAR-T in the exploratory analysis.

**Figure S2. Progression-free survival and overall survival of patients with extranodal disease who underwent CAR-T including those with secondary CNS lymphoma**


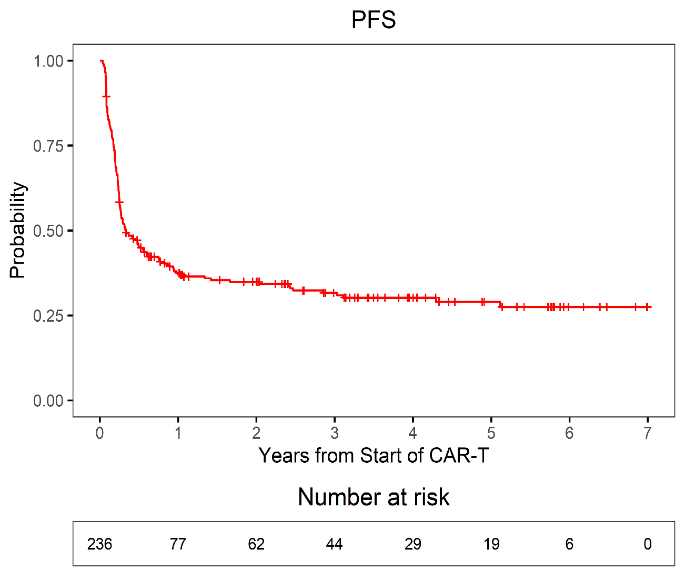

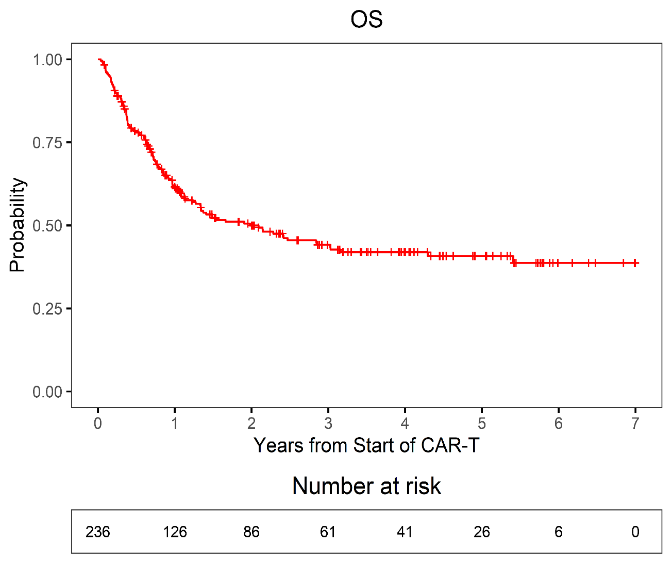


**Figure S3. Progression-free survival and overall survival by status of extranodal involvement at the time of CAR-T**

**
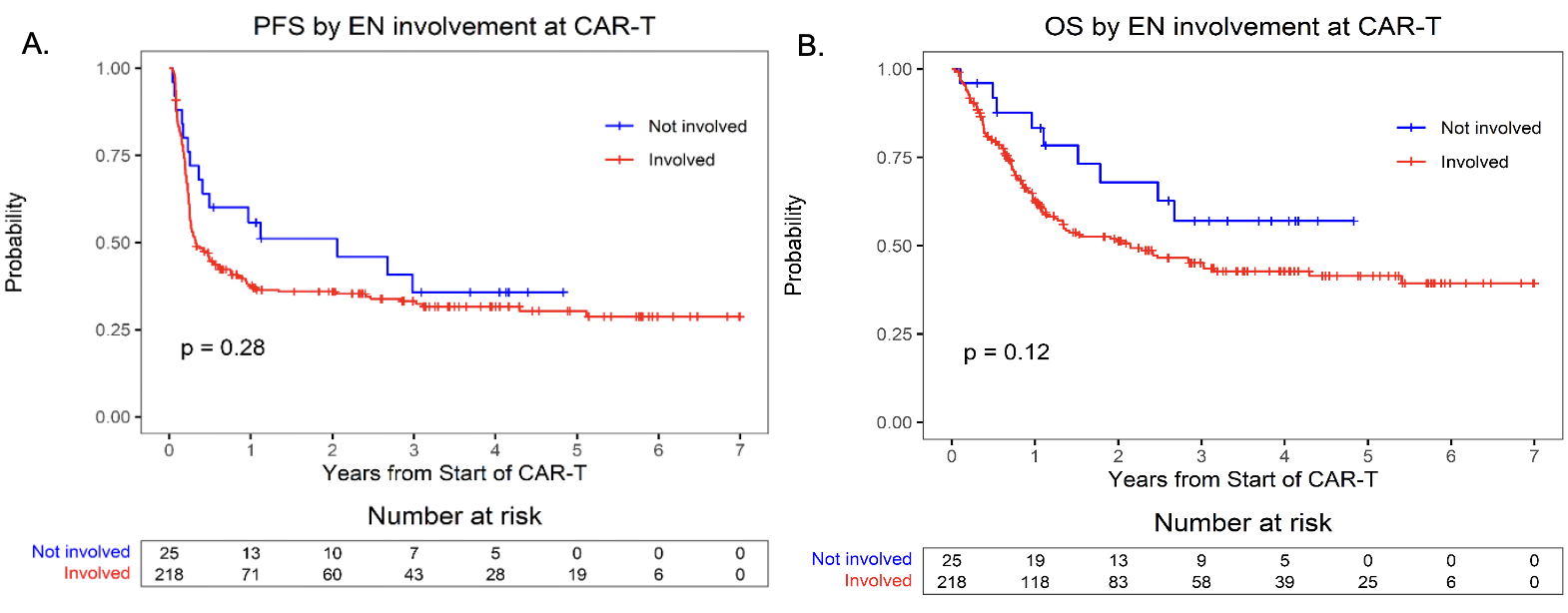
**

A: PFS by extranodal (EN) involvement at CAR-T. The KM estimates are shown for patients with (red curve) versus without (blue curve) EN involvement. There is no statistically significant difference between the curves (p=0.28). B: OS by EN involvement at CAR-T. The KM estimates are shown for patients with (red curve) versus without (blue curve) EN involvement. There is no statistically significant difference between the curves (p=0.12).
